# Objective Estimation of Frequency-Specific Pure-Tone Hearing Thresholds following Bone-Conduction Hearing Aid Stimulation

**DOI:** 10.1155/2014/247942

**Published:** 2014-04-10

**Authors:** Torsten Rahne, Thomas Ehelebe

**Affiliations:** Department of Otorhinolaryngology and Halle Hearing and Implant Center, University Hospital Halle (Saale) of Martin-Luther University Halle-Wittenberg, 06120 Halle (Saale), Germany

## Abstract

Patients suffering from conductive or mixed hearing loss may benefit from bone-conduction hearing systems (BAHS). The amount of amplification provided by the hearing system is selected based on the individual's sensorineural frequency-specific threshold. With patients who are not able to provide thresholds behaviorally, such as young children, objective methods are required to estimate the unaided and aided hearing threshold and thus the success of the hearing system fitting. In a prospective study with ten adult Baha softband users, aided and unaided frequency-specific thresholds were estimated. Aided thresholds to tone bursts via Baha stimulation were obtained behaviorally and electrophysiologically using cortical auditory evoked potentials (CAEPs) and were compared to pure-tone thresholds using routine clinical audiometry. For all stimulation frequencies, the frequency-specific electrophysiological and behavioral hearing thresholds measured with Baha stimulation were highly correlated and not different. Increased thresholds were observed only with the 0.5 kHz Baha stimulation as compared to the pure-tone audiogram. Objective measurement of frequency-specific hearing thresholds with CAEPs is applicable to BAHS users.

## 1. Introduction

Frequency-specific estimation of pure-tone hearing thresholds is used as behavioral measure in clinical routine [1]. As subjective responses to the presented pure-tones are required, this method is not applicable only to passively cooperative patients. With patients who are not able to provide thresholds behaviorally, such as young children or passively cooperative adults, objective methods are needed to estimate the hearing threshold. Therefore, auditory evoked potentials (AEP) are applicable [2–5]. Recording of cortical auditory evoked potentials (CAEPs) is the preferred method for the frequency-specific estimation of the hearing threshold [6]. Therefore, the lowest stimulation level where a P1-N1-P2 complex as a CAEP waveform with a latency of 50 to 175 ms can be discerned is usually defined as electrophysiological hearing threshold [2, 7]. As the amplitude and latency of the P1-N1-P2 waveform undergo significant maturation until the age of six years and the patients need to awake during the recording, this method is limited to juvenile and adult patients [8, 9]. The resulting electrophysiological thresholds correlate with the behavioral measured frequency-specific pure-tone thresholds [10–12].

CAEPs can also be reliably recorded in individuals when the acoustic stimulation is processed through a hearing aid [13, 14]. In this study, we apply the CAEP method to measure the hearing thresholds with adults using a Baha bone-anchored hearing system (Cochlear Ltd., Australia). With the Baha, a titanium screw is implanted to the temporal bone. After osseointegration, a percutaneously connected electromechanical sound processor transmits the sound transcranially via bone conduction to the cochlea fluids [15–17]. The sound processor needs to be fitted on the individual frequency-specific hearing loss. Therefore, the aided pure-tone audiogram serves as a control of the fitting [17–20].

Measuring hearing thresholds with Baha users objectively, the recording of auditory evoked potentials using Baha as stimulator was recently shown in normal hearing adults [21]. The behavioral threshold was reproduced with a difference of less than 10 dB hearing level (HL). Rahne et al. [21] used the Baha softband (Cochlear Ltd., Australia) for the acoustical stimulation and showed a CAEP threshold of 20 dB above the individual hearing threshold. A stimulation artifact caused by the electromagnetic vibror has been observed only with the recording of auditory brainstem potentials and stimulation levels above 50 dB normalized hearing level (nHL) and is thus not relevant to threshold estimation with CAEPs.

In this study, we measure the aided frequency-specific pure-tone hearing thresholds objectively by CAEP recordings with patients showing a sensorineural or mixed hearing loss. Therefore, electrophysiological thresholds were estimated with three audiometric frequencies and compared to the behaviorally measured aided and unaided thresholds.

## 2. Material and Methods

### 2.1. Subjects

Ten adults aging between 16 and 81 years (*M* = 62 years; three females, seven males) participated in the study. According to the WHO classification [22], eight participants had a mild and two participants had a moderate sensorineural hearing loss, in two cases combined with a conductive component of >15 dB in at least one frequency (see Figure 2). The participants gave informed consent after the procedures were explained to them, in accordance with the ethical guidelines of the Martin-Luther University of Halle-Wittenberg, where the study was conducted. The procedures conform to the code of ethics of the World Medical Association (declaration of Helsinki).

### 2.2. Experimental Setup and Procedure

The pure-tone hearing thresholds of the subjects were determined with routine clinical audiometry using sine tones with frequencies of 0.25, 0.5, 1, 1.5, 2, 3, 4, 6, and 8 kHz. The thresholds were determined separately for air (AC-PTA) and bone conduction (BC-PTA) using narrow band noise as masker if applicable. An SD50 audiometer (Siemens, Germany) with a TDH39 headphone (Telephonics, USA) for air-conduction stimulation and a KH70 transducer (Grahnert-Präcitronic, Germany) for bone-conduction stimulation were used which are established in the clinical routine. The better hearing side was determined by averaging the 0.5, 1, and 2 kHz bone-conduction thresholds [23].

For the bone-conduction hearing systems (BAHS) portion of the experiment tone bursts with frequencies of 0.5 kHz, 1 kHz, and 2 kHz were generated by the ESTIM2 signal generator and amplifier (ESMED, Germany) which also recorded the EEG. The stimulus length was 500 ms containing 15 ms of rise and fall time and its repetition rate was 0.5 Hz. The stimuli were delivered electrically to the external audio port of the Baha Intenso (Cochlear Ltd., Australia) whose amplification was not fitted to the individual hearing threshold. The vibrator was connected to the mastoid of the better hearing side using the Baha softband (Cochlear Ltd., Australia). The stimulation level was set by changing the amplitude level of the electrical signal provided by the signal generator. The program switch of the Baha Intenso was set to “external,” and the gain and tone controls were adjusted to its middle position which provided a linear I/O function of the Baha up to 60 dB SPL and a gain ranging from 55 dB (0.5 kHz and 2 kHz) to 60 dB (1 kHz). The volume switch of the Baha was adjusted to a value of “2.” The calibration of the stimuli was done subjectively. Therefore, the bone-conduction hearing thresholds for the tone bursts were determined behaviorally for the frequencies of 0.5 kHz, 1 kHz, and 2 kHz using the Baha as stimulator.

The electrophysiological estimation of the thresholds was done with the same setup. For the two-channel EEG recording Ag/AgCl electrodes were connected to the electrodes sites F3 (inverting) and the left mastoid (noninverting) and to F4 (inverting) and the right mastoid (noninverting). The gain was set to 100 *μ*V and the A/D rate was 400 Hz. The EEG data were filtered with a 15 Hz low pass. Prior to the averaging, the EEG was online epoched whereas whereas epochs with amplitudes reaching more than 95% of the maximal amplification range were rejected. The sound intensity level was initially set to 70 dB nHL and then reduced in steps of 5 dB until the individual electrophysiological threshold was estimated. At least, 90 artifact-free epochs were collected for every sound intensity level and averaged. The P1-N1-P2 waveforms and the lowest sound intensity level where a P1-N1-P2 waveform could be observed (CAEP threshold) were determined by visual inspection of the averaged waveforms by two audiologists. At every stimulation level, the recording was repeated. The P1 response was defined as the most prominent positive peak in the latency range of 0–80 ms; the N1 response was defined as the most prominent negative peak in the latency range of 50–150 ms; the P2 response was defined as the most prominent positive peak in the latency range of 100–200 ms. The absence of the response was stated if there was no reproducible P1, N1, and P2 responses in both recordings. For all conditions, the subjects were comfortably seated in a sound attenuated booth. The total duration of the experiment including breaks was about 1.5 hours.

### 2.3. Data Analysis

The individual Baha hearing thresholds were compared by a two-way ANOVA for repeated measures using the factors frequency (1, 2, and 3 kHz) and method (behavioral and electrophysiological). The assumption of sphericity for the repeated measures variables was tested with the Mauchly test. Greenhouse-Geisser corrections for violations of sphericity were applied when necessary. Post hoc comparisons were made with Fisher's least significant difference test (LSD). *α* was set to 95% for all comparisons. Regression analysis was done between the behavioral and electrophysiological thresholds for all frequencies. The electrophysiological hearing thresholds and the behavioral pure-tone bone-conduction threshold measured with the routine clinical audiometry were compared descriptively.

## 3. Results

**Figure 1:**
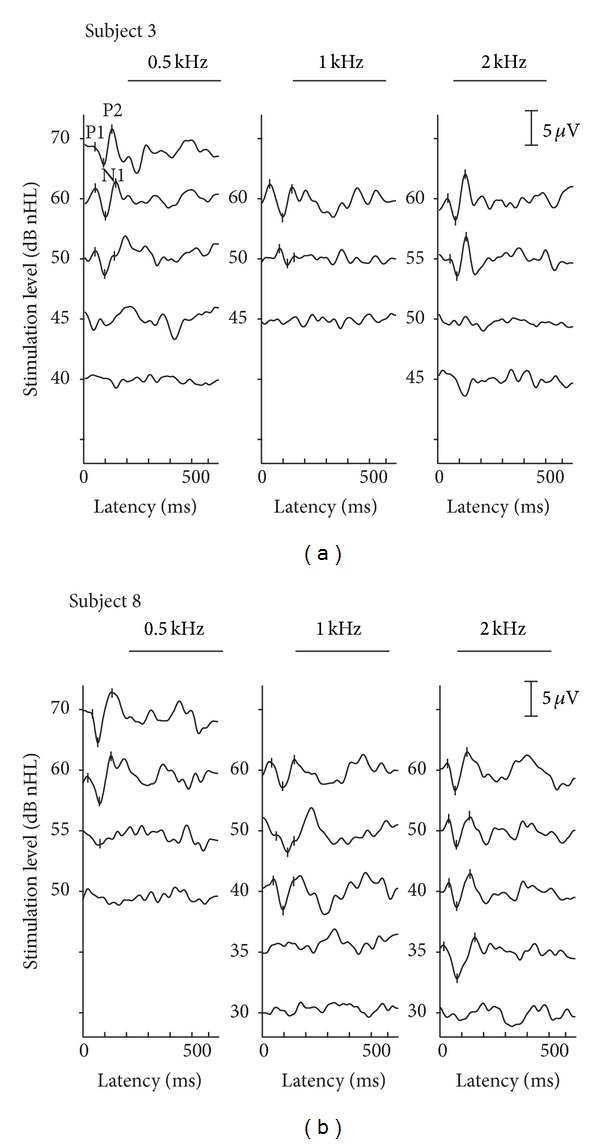
CAEP average waveforms recorded from the exemplary subjects 3 (a) and 8 (b) using a Baha Intenso with a softband as transducer. P1-N1-P2 waveforms are marked for the stimulation frequencies of 0.5 kHz, 1 kHz, and 2 kHz if present. The lowest stimulation level where the P1-N1-P2 waveform was discernible was defined as electrophysiological threshold.

**Figure 2:**
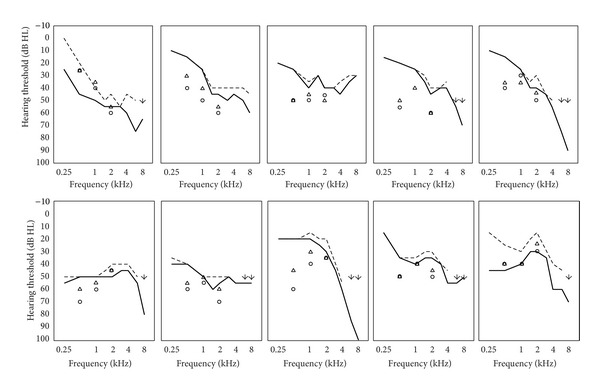
Individual audiograms showing pure-tone air-conduction (thick line) and bone-conduction (dotted line) audiograms measured with routine clinical audiometry. For every subject, the side with the best bone-conduction PTA threshold is shown. Arrows mark threshold exceeding the maximum output limit of the bone-conduction transducer at 6 kHz and 8 kHz. Aided behaviorally (triangles) and electrophysiologically (circles) measured pure-tone thresholds for 0.5 kHz, 1 kHz, and 2 kHz Baha stimulation were also shown using the same Baha setting for all subjects.

Figure 1 displays the recording of the CAEPs for two exemplary subjects. The P1-N1-P2 waveforms were discernible and no artifacts caused by the Baha as described by Rahne et al. [21] were observed. The P1-N1-P2 amplitudes decreased with decreasing stimulation level. The resulting electrophysiological thresholds as well as the behaviorally determined pure-tone thresholds with the Baha transducer are shown in Figure 2. The PTA results show a sensorineural hearing loss of different degrees with partially an additional conductive component. With stimulation frequencies of 6 kHz and 8 kHz, the maximum output limit of the bone-conduction transducer of 50 dB HL was exceeded in 5 patients. The difference between the electrophysiological CAEP thresholds and the bone-conduction PTA thresholds ranges from 5 to 40 dB (*M*: 22.5 dB) for the 0.5 kHz stimulation, from 0 to 25 dB (*M*: 11.1 dB) for the 1 kHz stimulation, and from 5 to 20 dB (*M*: 16.5 dB) for the 2 kHz stimulation.

**Figure 3:**
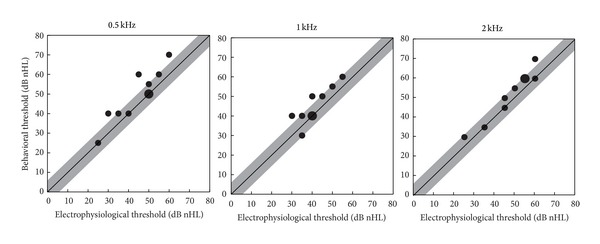
Individual behavioral pure-tone threshold for all subjects as function of the electrophysiologically measured threshold, for both stimulation with the Baha as transducer and frequencies of 0.5 kHz, 1 kHz, and 2 kHz shown as bubble chart (small circle: *n* = 1, large circle: *n* = 2). Linear regression analysis was significant for all frequencies. Shaded areas mark the difference of ±5 dB from the diagonal which was the resolution of the threshold measurements.

Figure 3 shows the behavioral pure-tone threshold with a function of the electrophysiological threshold, both measured with the Baha transducer. The thresholds were significantly different between the methods (ANOVA, *F*(1,9) = 23.3, *P* < 0.01). No difference between the frequencies or interaction of the frequency and the method was observed. The mean difference between the behavioral and electrophysiological Baha thresholds (behavioral-electrophysiological offset) was 4.4 dB (SD: 0.9 dB) which is below the resolution of the threshold determination (5 dB). A regression analysis showed a significant linear regression between the electrophysiological and the behavioral thresholds for 0.5 kHz (*r* = 0.91, *P* < 0.001), 1 kHz (*r* = 0.83, *P* < 0.01), and 2 kHz (*r* = 0.97, *P* < 0.001).

## 4. Discussion

In this study, we measured CAEPs when auditory stimulation was applied via a Baha transducer. With this, electrophysiological pure-tone thresholds were measured frequency-specifically for a group of subjects with different behavioral bone-conduction thresholds. For all stimulation frequencies used, clear P1-N1-P2 waveforms could be discerned. The latencies and amplitudes are comparable to those evoked with headphone stimulation [2, 4, 7]. The reduction of the stimulation level caused decrease in P1-N1-P2 amplitudes and thus the estimation of the electrophysiological threshold was possible with all subjects.

CAEP thresholds would be helpful to control the fitting of the Baha processor objectively. As in this study the Baha processor was not fitted to the individuals' PTA, the electrophysiological thresholds cannot be compared to the behavioral bone-conduction thresholds. For that reason, the behavioral pure-tone thresholds were also measured by Baha stimulation using the same acoustical stimulation setup as used for the CAEP recording. For all stimulation frequencies used, the electrophysiological and behavioral pure-tone thresholds show a good correlation with almost no behavioral-electrophysiological offset as described by previous measurements [24–26]. In particular, due to difficulties in eliciting CAEPs with low frequencies, a large offset was expected [27]. Considering all stimulation frequencies used the behavioral-electrophysiological offset in this study was the largest with the 0.5 kHz stimulation. However, even this offset is smaller than the resolution of the electrophysiological threshold estimation and also smaller than the previously reported values [24–26].

Recording CAEPs using a Baha as transducer could interfere with its unknown signal processing of the stimulus and artifacts in the EEG recording caused by the electromagnetic transducer as described by Rahne et al. [21] for auditory evoked brainstem potentials. However, for all stimulation frequencies and sound intensity levels used, no stimulation artifact was observed which allowed a reliable determination of the P1-N1-P2 waveform even close to the electrophysiological threshold. Probably the low-pass cutoff of the EEG would have omitted the artifacts which are mainly generated as stimulus-on effect [21] and should be applied for further recordings.

Obviously, the electrophysiological threshold did not coincide with the bone-conduction threshold measured behaviorally with the routine clinical audiometry. This was expected because the Baha was temporarily fitted to the hearing impaired subject's skin by a softband. It is known that threshold shifts of 5 to 20 dB occur if transcutaneous and percutaneous coupling of Baha devices are compared [28, 29]. That large variance almost completely covers the threshold difference between the behavioral Baha thresholds and the routine clinical audiometry in this study. However, the insufficient amplification of the unspecific fitted sound processor also contributed to the absolute difference between the thresholds. When using CAEP recording to estimate hearing thresholds with individuals using an implanted Baha, the acoustic stimuli have to be calibrated to the individual hearing level. Then, the results could be applied with the clinical control of the Baha fitting, especially with only passively cooperative patients. If additionally the different morphology of the P1-N1-P2 between adults and children is considered [8], also the Baha fitting of children could profit from the results. Also other clinical applications as the fitting of the bonebridge or the evaluation of speech perception with hearing aids [7, 30] could profit from the results if the calibration of the signals and the respective signal processing are adapted in the respective devices.
